# Case of Hydrocephalus Successfully Treated by the Removal of the Water by Operation

**Published:** 1819-07-01

**Authors:** 


### *Case of Hydrocephalus successfully treated by the Removal of the Water by Operation.* By James Vose, M. D.

(Medico-Chirurgical Transactions, vol. ix.)

The patient was an infant of seven weeks old, whose head was enlarged by the accumulated fluid to between two and three times its natural size. There was but little ossification at birth, very soon after which, the mother perceived the head to increase in size, which had gone on progressively. The head was now become so transparent that when held between the eye and the light, it was not inaptly compared to a paper lantern. The child at the time first visited, was free from any symptoms indicative of a serious affection of the general health, with the exception of a slight derangement of the bowels, and occasional convulsions. Three ounces and five drachms of a limpid fluid were drawn off by puncture with a common couching needle, and the opening was closed by adhesive plaster and roller. About an equal quantity dribbled from the orifice after the operation, and the child sunk so extremely low as to create the greatest alarm. Revival, however, took place, without the aid of medicine, and the water having again accumulated, the operation was repeated, with less precaution, the puncture being performed with the curved and pointed bistoury; five ounces were drawn off without any unpleasant accident following. This operation was repeated twice or thrice afterwards, and the child ultimately recovered. It is remarkable that after the last operation, there was a considerable discharge of water from the bowels, very similar to that drawn off from the head. After this, ossification advanced with great rapidity; the child improved in health, size, and vigour. Its appearance became good, and no convulsions afterwards occurred.

